# Cerebral ischemia-induced angiogenesis is dependent on tumor necrosis factor receptor 1-mediated upregulation of α5β1 and αVβ3 integrins

**DOI:** 10.1186/s12974-016-0697-1

**Published:** 2016-09-01

**Authors:** Heng Huang, Qijuan Huang, Fuxin Wang, Richard Milner, Longxuan Li

**Affiliations:** 1 Department of Neurology, Gongli Hospital, 219 Miaopu Road, Pudong New Area, Shanghai, 200135 People’s Republic of China; 2 Department of Neurology, Guangdong Medical University Affiliated Hospital, Zhanjiang, 524001 People’s Republic of China; 3 Department of Molecular and Experimental Medicine, The Scripps Research Institute, 10550 North Torrey Pines Road, La Jolla, CA 92037 USA

**Keywords:** Angiogenesis, Cerebral ischemia, Integrin, Tumor necrosis factor-α

## Abstract

**Background:**

The pro-inflammatory cytokine, tumor necrosis factor-α (TNF-α), is expressed in ischemic tissue and is known to modulate angiogenesis; however, the role of the two distinct TNF-α receptors, TNFR1 and TNFR2, in mediating angiogenic signaling after cerebral ischemic stroke is relatively unknown.

**Methods:**

C57BL6 mice were subject to 90 min of ischemia by temporary occlusion of the middle cerebral artery (MCAO) and given daily intra-cerebroventricular injections of antibodies against TNFR1, TNFR2 or control IgG (doses of 10, 50, and 100 ng/day) for 4 days following 90 min MCAO. Vascular remodeling and α5β1 and αVβ3 integrin expression were then examined in the brains of these mice after 4, 7, and 14 days post-ischemia. In parallel in vitro studies, flow cytometry was used to determine the influence of TNF-α on proliferation and integrin expression of human brain microvascular endothelial cells (HBMECs).

**Results:**

The post-ischemic cerebral angiogenic response was inhibited by antibodies against TNFR1 but not TNFR2, and this correlated with reduced endothelial proliferation and decreased α5β1 and αVβ3 integrin expression after 4 and 7 days post-ischemia. Consistent with these findings, in vitro studies showed that TNF-α induced endothelial proliferation and upregulation of α5β1 and αVβ3 integrins was abrogated by anti-TNFR1 but not anti-TNFR2 antibodies in cultured HBMECs. In addition, blocking antibodies to α5β1 and αVβ3 integrins significantly inhibited TNF-α-induced HBMEC proliferation.

**Conclusions:**

Our results suggest that TNFR1-mediated signaling plays a critical role in triggering angiogenic integrins and subsequent angiogenic responses following cerebral ischemia. These novel findings could form a platform for future therapeutic strategies aimed at stimulating angiogenesis following cerebral ischemia.

**Electronic supplementary material:**

The online version of this article (doi:10.1186/s12974-016-0697-1) contains supplementary material, which is available to authorized users.

## Background

Growing evidence underlines the therapeutic potential of angiogenic processes post-stroke. Greater microvessel density in the ischemic border correlates with longer survival in stroke patients [1], and conversely, older patients who do much worse after stroke compared to younger patients [2, 3] have reduced new vessel formation [4]. These results suggest that angiogenic remodeling may improve cerebral perfusion and function as part of a coordinated repair response. In a previous study using a mouse model of focal cerebral ischemia, we demonstrated the presence of proliferating endothelial cells in the ischemic penumbra and found that this correlated with increased vessel density [5].

More recently, we described that in response to cerebral ischemia, vascular expression of fibronectin (Fn), and its two receptors, α5β1 and αVβ3 integrins, were all noticeably increased by day 4, peaked at day 7, then declined at day 14. Interestingly, brain endothelial cell (BEC) proliferation followed the same time-course, suggesting a mechanistic relationship, with the Fn-α5β1/αVβ3 integrin axis driving BEC proliferation and angiogenesis. This is consistent with our previous work in the chronic hypoxia model in which transgenic mice lacking endothelial expression of α5 integrin (α5-EC-KO mice) showed a delayed and reduced angiogenic response to mild hypoxia [6].

Interestingly, angiogenic vessels in the ischemic penumbra are often surrounded by inflammatory microglia and macrophages [7–9], suggesting that activated microglia and/or macrophages may be instrumental in promoting the angiogenic response to cerebral ischemia. To directly examine this question, in a previous study, we exposed BEC to microglia-conditioned media in vitro and found that a microglia-conditioned medium from activated microglia directly promoted BEC proliferation, and these effects are largely attributable to the microglial cytokine TNF-α. In addition, pure TNF-α promoted BEC proliferation in a dose-dependent manner [10].

Known as a strong immune-mediator and pro-inflammatory cytokine, TNF-α is rapidly upregulated in the brain after injury [11]. TNF-α interacts with two types of cell surface receptors, TNF receptor 1 (TNFR1) and TNFR2 [12]. TNFR1 is expressed on all cell types, and this is a major signaling receptor for TNF-α, whereas TNFR2 is expressed primarily by endothelial cells and hematopoietic and some neuronal populations and mediates limited biological responses [12].

TNFR1 primarily mediates apoptosis and inflammation [13] but has also been shown to have anti-apoptotic functions via activation of the NF-kB signaling pathway [14–16]. In the context of stroke, TNFR1 has been shown to mediate ischemic tolerance [17] and neuroprotection [18, 19] presumably through the TNFR1-NF-kB-FLIP L pathway [20]. After permanent focal cerebral ischemia, TNFR1-KO mice developed significantly larger infarct volumes compared with TNFR2-KO and wild-type mice, suggesting that TNF-α exerts neuroprotective effects through TNFR1 [18]. Furthermore, the interaction of TNF-α with TNFR1 sensitizes cerebral endothelial cells to erythropoietin-induced angiogenesis [21]. Neuroprotection through TNFR2 has also been reported [22, 23]. In addition, TNFR1 and TNFR2 have been reported to play differential roles in hindlimb ischemia-mediated arteriogenesis and angiogenesis, with TNFR1 inhibiting, but TNFR2 signaling promoting, hindlimb ischemic-induced arteriogenesis and angiogenesis [24]. The exact mechanisms underlying such different properties of TNF-α receptors have yet to be fully determined, but these might be explained by intrinsic differences in endothelial cells between different organs and different pathophysiological conditions.

In rodent models of permanent focal cerebral ischemia, TNF messenger RNA (mRNA) and protein levels are elevated 4–6 h after the ischemic insult [11, 25–27]. In our earlier study of transient focal cerebral ischemia, we noted that peak levels of TNF-α in the ischemic brain precedes that of α5β1 and αvβ3 upregulation, and our in vitro study showed that TNF-α stimulation promoted strong upregulated expression of α5β1 and αVβ3 integrins on human brain microvascular endothelial cells (HBMECs) [28]. This suggests that TNF-α induction post-cerebral ischemia may be instrumental in promoting expression of the α5β1 and αvβ3 integrins as part of the angiogenic process. In light of these outstanding questions, the goal of the current study was to determine which TNF-α receptor is involved in promoting post-ischemic angiogenesis and whether TNF-α-α5β1/αVβ3 signaling is required for effective ischemia-induced angiogenesis.

## Methods

### Experimental animals

Male C57Bl/6 mice weighing 20–25 g at the time of surgery were used for all experiments. The present study was conducted in accordance with NIH guidelines for the care and use of animals in research and under protocols approved by the Animal Care and Use Committee of Gongli Hospital, Pudong New Area, Shanghai.

### Surgical procedures

Under anesthesia, mice were placed in a stereotaxic frame, and a 24-G stainless steel guide cannula was implanted 1 mm above the lateral cerebral ventricle and fixed in place with dental acrylic, as described previously [29]. After 1 day recovery, mice were anesthetized with pentobarbital and underwent 90 min right middle cerebral artery occlusion (MCAO) followed by reperfusion as described previously [9]. Anti-mouseTNFR1 (R&D Systems, Minneapolis, MN, USA) or TNFR2 (BioLegend, San Diego, CA, USA) monoclonal antibody (each in doses of 10, 50, and 100 ng/day) was given intracerebroventricularly (i.c.v.) through a pre-implanted guide cannula into the right lateral cerebral ventricles within 5 min after 90 min MCAO while animals were still anesthetized. Each mouse received daily i.c.v injections consecutively for 4 days of reperfusion following 90 min MCAO. As a control, purified hamster IgG alone was injected into the lateral ventricle of each mouse. Accurate placement of the injection or needle track was verified at the time of dissection. Mice were euthanized 4, 7, or 14 days post-ischemia.

### Immunofluorescent studies and antibodies

Mice at different time-points of reperfusion were euthanized by perfusion with ice-cold saline, and the brains rapidly dissected and stored at −80 °C. As we previously described [5], TTC staining was performed to demarcate the cerebral infarct and ischemic penumbra. Immunofluorescent (IF) studies were performed as previously described [30] on 10-μm-thick frozen coronal sections. The following monoclonal antibodies from BD Pharmingen (La Jolla, CA, USA) were used in this study: FITC-conjugated rat anti-mouse CD31 (PECAM-1) (clone MEC13.3; 1:100) and PE-conjugated rat anti-mouse α5(CD49e) (clone 5H10-27; 1:100). The rabbit anti-fibronectin (F3648) was purchased from Sigma (St. Louis, MO, USA; 1:400). The rabbit anti-Ki67 (ab15580) was obtained from Abcam (Cambridge, MA, USA; 1:800). The rabbit anti-CD31 (PECAM-1) (250590) antibody was obtained from Abbiotec (San Diego, CA, USA; 1:200). The rabbit anti-integrin β3 (MBS8508681) antibody was purchased from MyBioSource (San Diego, CA, USA; 1:200). The Alexa Fluor 488-conjugated goat anti-rat and anti-rabbit, and Cy-3-conjugated goat anti-rat and anti-rabbit secondary antibodies were obtained from EarthOx (Millbrae, CA, USA).

### Cell culture

Primary human brain microvascular endothelial cells (HBMECs) were purchased from Cell Systems (Kirkland, WA, USA). Cells were grown on six-well plates pre-coated with type I collagen (10 μg/ml, Sigma, for 2 h at 37 °C). The culture medium was endothelial basal medium (EBM-2) (Lonza, CC-3156) supplemented with 10 % FBS (Gibco), ascorbic acid, l-glutamine, penicillin/streptomycin, and human basic fibroblast growth factor (bFGF) (all from Sigma). Cells were maintained in a humidified incubator at 37 °C and 5 % CO_2_, and the medium was changed every 48 h.

### CFSE staining and cell proliferation

As previously described [31], HBMECs (10^7^ cells/ml) were labeled with carboxy-fluorescein succinimidyl ester (CFSE; BD Biosciences, San Jose, CA, USA), for 10 min at 37 °C with 5 μM CFSE in serum-free RPMI 1640 medium. The labeling reaction was stopped by the addition of ice-cold complete RPMI 1640 medium at 4 °C for 5 min, followed by centrifugation at 1000 rpm for 5 min. To assess cell proliferation, 2 × 10^4^ CFSE-labeled HBMECs were seeded in six-well plates pre-coated with type I collagen (Sigma) and cultured until ~50 % confluent. For investigation of TNF-α, recombinant human TNF (rhTNF)-α (R&D Systems, Minneapolis, MN, USA) was added to the HBMEC cultures to a final concentration of 10 ng/ml. The same volume of saline with DMSO vehicle was used as control. To inhibit TNF-α receptor function, neutralizing antibodies (R&D Systems) to TNFR1 or TNFR2 (BioLegend, San Diego, CA, USA) were added to the HBMEC cultures at 10 μg/ml for 30 min prior to stimulation with rhTNF-α. To examine the role of specific integrins in promoting HBMEC proliferation, the HBMECs were pre-incubated with mouse anti-human integrin α5, αVβ3 (both from Millipore, Temecula, CA, USA), or a combination of both monoclonal antibodies at 5 μg/ml for 30 min prior to stimulation with rhTNF-α. The co-cultures were incubated for 24 h. The cells were then harvested, washed twice, re-suspended in FACS buffer, and analyzed using a FACS Canto II flow cytometry (FCM; BD, USA) by acquiring a minimum of 20,000 events from each sample. The cells were excited by a 488-nm argon ion laser and analyzed at 530 nm (showing CFSE). The flow cytometry data files were analyzed using the Proliferation Wizard module of the ModFit software (Verity Software House, USA). The proliferation index was calculated as the sum of cells in all generations divided by the number of original parent cells and expressed as the percentage of the control.

### Determination of integrin expression by flow cytometry

Confluent HBMECs were cultured in collagen-coated six-well plates and treated with or without rhTNF-α (R&D Systems) at 10 ng/ml for 24 h before flow cytometry analysis. To determine the effects of blocking antibodies on HBMEC integrin expression, neutralizing antibodies to TNFR1 or TNFR2 were added to the HBMEC cultures at 10 μg/ml for 30 min prior to stimulation with rhTNF. Integrin expression of HBMECs was examined as described previously [32]. Briefly, cells were labeled with anti-human α5-PE (BD), anti-human β1-PE (BD), anti-human αV-PE (Abcam, Cambridge, MA, USA) or anti-human β3-PE (eBioscience, San Diego, CA, USA). Mouse IgG1-PE isotype (Abcam) or k-PE isotype (eBioscience) was used as control antibody. Samples were analyzed by flow cytometry, and the mean fluorescent intensity (MFI) of labeled cells was analyzed by Cell Quest software. Expression level of each integrin subunit was shown as a percent change from the control value.

### Quantification and statistical analysis

Quantification of the number of blood vessels positive for the different antigens was performed by capturing images of the region of interest (ischemic penumbra including the cortex and striatum) with a ×20 objective on a Leica TCS SP5 II microscope. These images were used to determine the number of positive events per field of view (FOV). For each antigen, three brain sections were taken from each animal and matched between animals so that the approximate position of sections used was equivalent between different experimental conditions. Three images were taken from cortex and striatum of each brain section and quantified by eye for the number of positive events per FOV. The total number of antigen-positive events per FOV in each of regions (cortex and striatum) was averaged together to represent the number for each section. These averages of three brain sections were used for statistical analysis for each animal. Each experiment was performed with five different animals per condition, and the results expressed as the mean ± SEM of the number of antigen-positive event per FOV. The in vitro assays of cell proliferation and flow cytometry were performed in duplicate a minimum number of four times, and the results presented as the mean ± SEM. Statistical significance was assessed by *t* test or repeated-measures of two-way analyses of variance (ANOVA); and a Bonferroni post hoc test was used to test multiple comparisons. All statistical analyses were performed in SPSS (version 16.0; SPSS, Chicago, IL, USA), and the significance was defined as *P* < 0.05.

## Results

### Blocking TNFR1 function early following cerebral ischemia reduces angiogenic remodeling in the ischemic penumbra

In this study, we employed the mouse model of temporary focal cerebral ischemia, in which, C57BL/6 mice are subject to 90 min of ischemia by temporary occlusion of the middle cerebral artery (MCAO). As previous work has shown that TNF-α mRNA and protein levels are elevated 4–6 h after ischemic insult [11, 25–27] and peak between days 1 and 2 [28], we administered daily i.c.v. injections of neutralizing antibodies against TNFR1 or TNFR2 or control IgG (at doses of 10, 50, and 100 ng/day) for the first 4 days following 90 min MCAO, to clarify which TNF receptor is mediating post-ischemic angiogenesis.

After 4 days i.c.v. injections, some of the stroke mice were euthanized to do TTC staining. At the lowest antibody concentration (10 ng/day), there was no significant difference between infarction volumes in TNFR1, TNFR2 antibody-injected mice, or the control group. However, at higher antibody doses (50 and 100 ng/day), mice receiving TNFR1 antibody showed larger infarct size than mice receiving TNFR2 antibody or control antibody. This finding is consistent with the study of Lambertsen KL et al., who reported that after permanent focal cerebral ischemia, TNFR1-KO mice developed significantly larger infarct volumes compared with TNFR2-KO and wild-type mice [18].

**Fig. 1:**
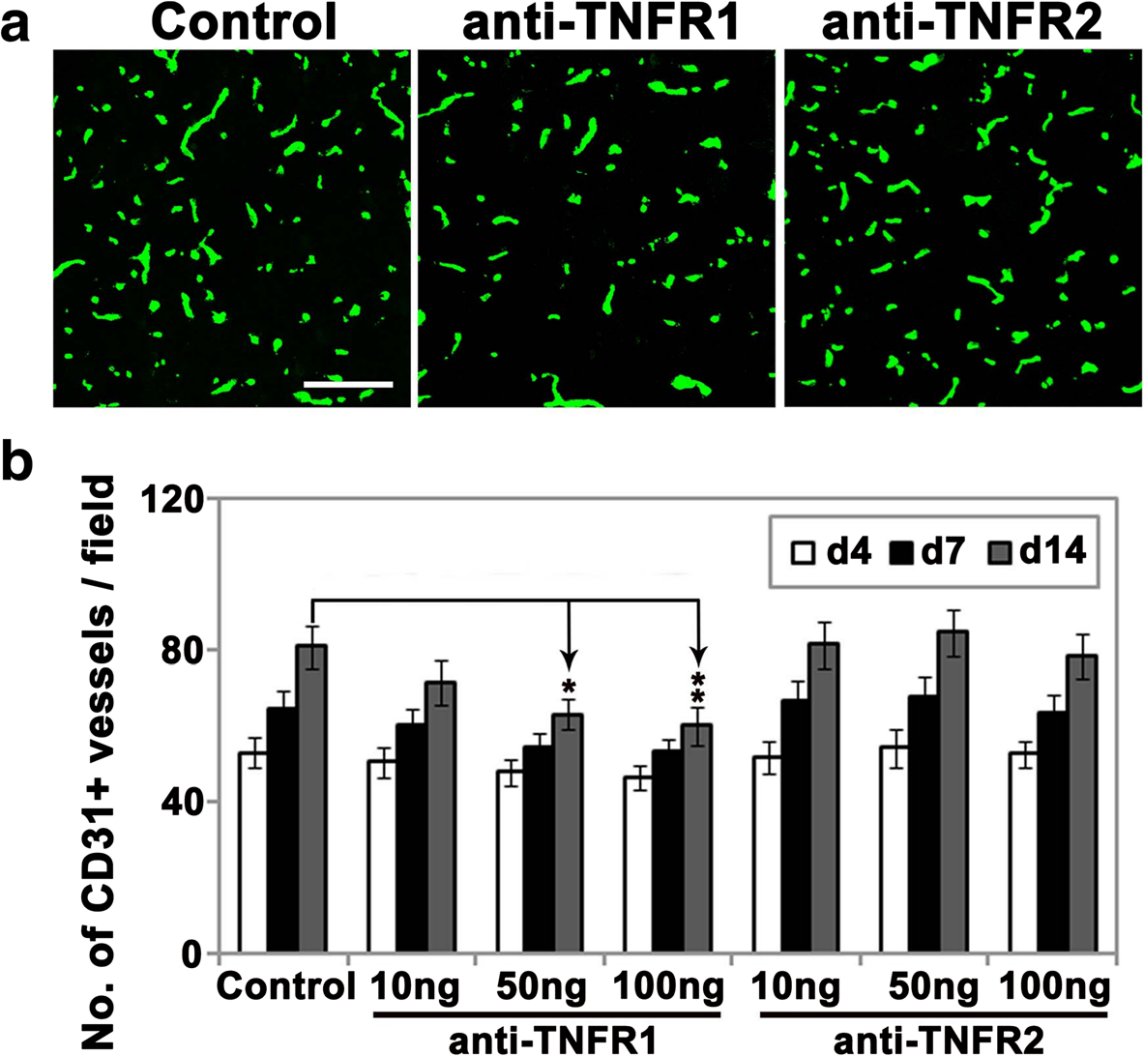
Pharmacological blockade of TNFR1, but not TNFR2, reduced the angiogenic response in the cerebral ischemic penumbra. Frozen sections of ischemic penumbra taken from mice after 4, 7, or 14 days reperfusion following 90 min MCAO and having received daily i.c.v. injections of antibodies against TNFR1 or TNFR2 or control IgG, were stained for the endothelial-specific marker CD31 (AlexaFluor-488). **a** Daily injection TNFR1 antibody (50 ng/day), but not TNFR2 antibody, noticeably reduced CD31 positive blood vessel density in the ischemic penumbra compared with controls over the 14 days following MCAO. *Scale bar* = 100 μm. **b** Quantification of CD31+ vessels, in which each experiment was performed with five different animals at different time-points post-ischemia. The results are expressed as the mean ± SEM of the number of CD31+ vessels per FOV. Note that the anti-TNFR1 antibody (at doses of 50 and 100 ng/day), but not the anti-TNFR2 antibody, significantly reduced post-ischemic increases in vessel density in the ischemic penumbra following 14 days reperfusion. *P < 0.05, **P < 0.01 versus control

As our previous work showed that BECs start to proliferate in the ischemic penumbra as early as 1 day post-ischemia and reach a maximum mitotic level at day 7, before declining at day 14, but that the newly formed vessels persist at least until day 14 post-ischemia [9], we looked for differences in the angiogenic response at 4, 7, and 14 days following MCAO. Using CD31 labeling as an index of blood vessel density, we found that daily injection of TNFR1 antibody (50 and 100 ng/day), but not TNFR2 antibody, noticeably reduced blood vessel density in the ischemic penumbra compared with controls over the 14 days following MCAO (Fig. 1a).

According to the two-way repeated-measures ANOVA, the effect of treatment (antibodies against TNFR1, TNFR2, or control IgG) (DF = 6; *F* = 5.06; *P* < 0.001) and time (day 4, 7, or 14) (DF = 2; *F* = 44.00; *P* < 0.001) were highly significant. The interaction of the variables “time and treatment” was not significant (DF = 12; *F =* 0.54; *P =* 0.88). The Bonferroni post hoc test revealed that blood vessel density (CD31-positive vessels per FOV) in the ischemic penumbra in TNFR1 antibody-injected mice (50 and 100 ng/day for 4 days) was significantly less than that of controls during the 14 days reperfusion following MCAO (50 ng group: 63.1 ± 4 vs. 80.9 ± 5.7, *P <* 0.05; 100 ng group: 60.1 ± 5 vs. 80.9 ± 5.7, *P <* 0.01). However, the TNFR2 antibody had no appreciable effect on vessel density. These studies suggest that TNFR1 but not TNFR2 plays a role in mediating the angiogenic response after cerebral ischemia.

### The mitogenic response to cerebral ischemia is mediated through TNFR1 not TNFR2

**Fig. 2:**
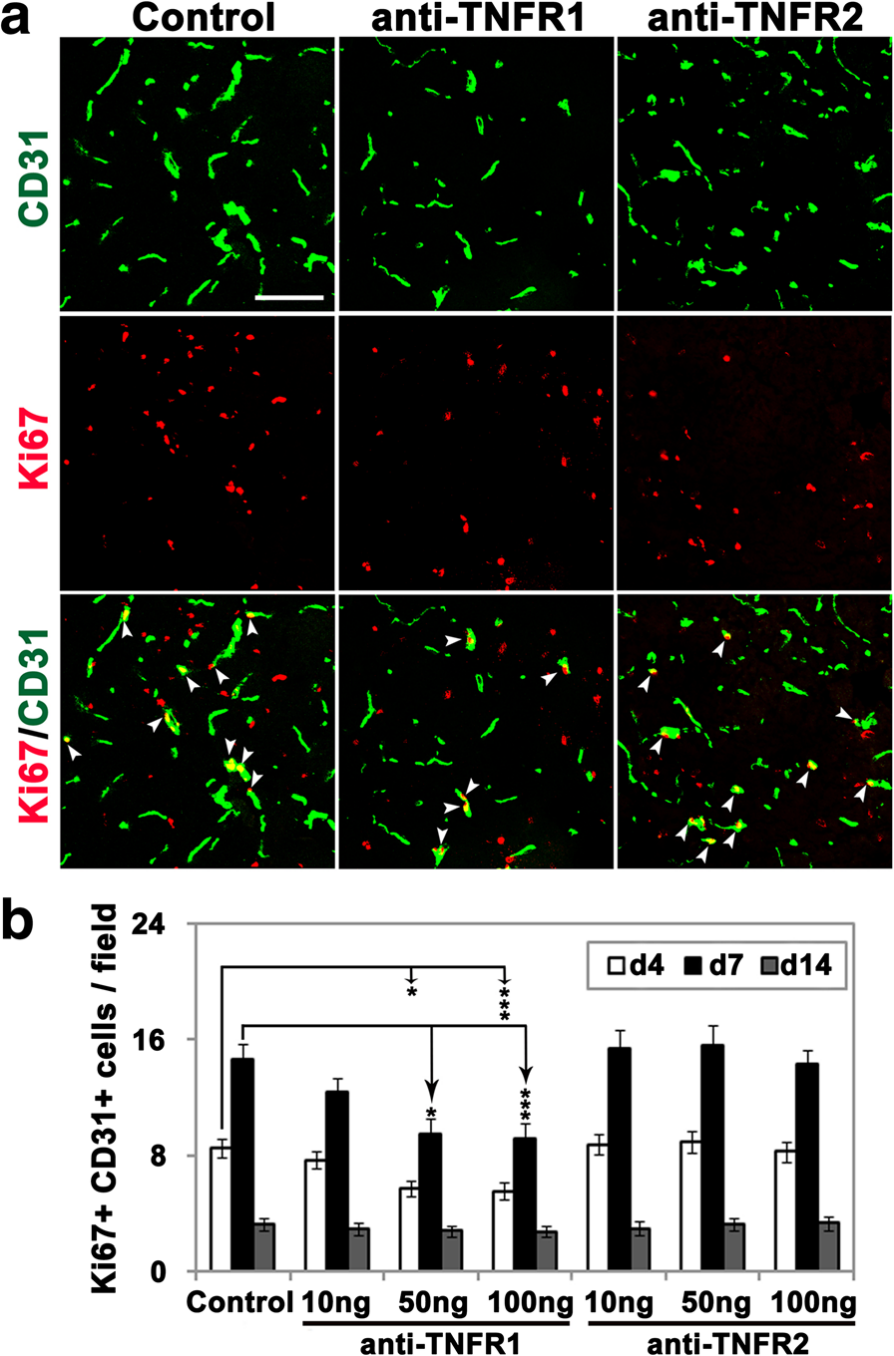
Pharmacological blockade of TNFR1, but not TNFR2, reduced endothelial cell proliferation in the cerebral ischemic penumbra. **a** Frozen sections of ischemic penumbra taken from mice after 4, 7, or 14 days reperfusion following 90 min MCAO and having received daily i.c.v. injections of antibodies against TNFR1 or TNFR2 or control IgG, were dual-stained for CD31 (AlexaFluor-488) and the proliferation marker Ki67 (Cy-3). *Scale bar* = 100 μm. **b** Quantification of CD31/Ki67 (*white arrowheads* in **a**) dual-positive proliferating BECs, in which each experiment was performed with five different animals at different time-points post-ischemia. The results are expressed as the mean ± SEM of the number of CD31/Ki67 dual-positive cells per FOV. Note that the anti-TNFR1 antibody (at doses of 50 and 100 ng/day), but not the anti-TNFR2 antibody, resulted in significantly reduced endothelial proliferation in the penumbra at 4 and 7 days post-ischemia. *P < 0.05, ***P < 0.001 versus control

We next examined whether the effect of the TNFR1 antibody in reducing vessel density in the post-ischemic penumbra might be a result of reduced endothelial proliferation. According to the two-way repeated-measures ANOVA, the effect of treatment (antibodies against TNFR1, TNFR2, or control IgG) (DF = 6; *F =* 10.21; *P <* 0.001) and time (day 4, 7 or 14) (DF = 2; *F =* 282.40; *P <* 0.001) were highly significant. The interaction of the variables “time and treatment” was also significant (DF = 12; *F =* 2.61; *P <* 0.01). As shown in Fig. 2, the Bonferroni post hoc test showed that after 4 or 7 days reperfusion following 90 min MCAO, the number of dual-positive Ki67+/CD31+ cells per FOV in the ischemic penumbra of TNFR1 antibody-injected mice (50 and 100 ng/day groups) was significantly lower than in control mice (50 ng group: 5.7 ± 0.6 vs. 8.5 ± 0.6, *P <* 0.05 at day 4, 9.5 ± 1.0 vs. 14.6 ± 1.1, *P <* 0.001 at day 7; 100 ng group: 5.5 ± 0.6 vs. 8.5 ± 0.6, *P <* 0.05 at day 4, 9.1 ± 1.1 vs. 14.6 ± 1.1, *P <* 0.001 at day 7). The number of Ki67+/CD31+ positive events under all conditions had reduced to almost baseline levels 14 days following reperfusion. In contrast to the TNFR1 antibody, the TNFR2 antibody had no appreciable effect on the number of Ki67+/CD31+-positive cells. These results demonstrate that the TNFR1 antibody reduced the endothelial mitogenic response to cerebral ischemia, suggesting a role for TNFR1 but not TNFR2 is mediating ischemic-induced endothelial proliferation.

### TNF-α promotes brain endothelial proliferation via TNFR1 not TNFR2

**Fig. 3:**
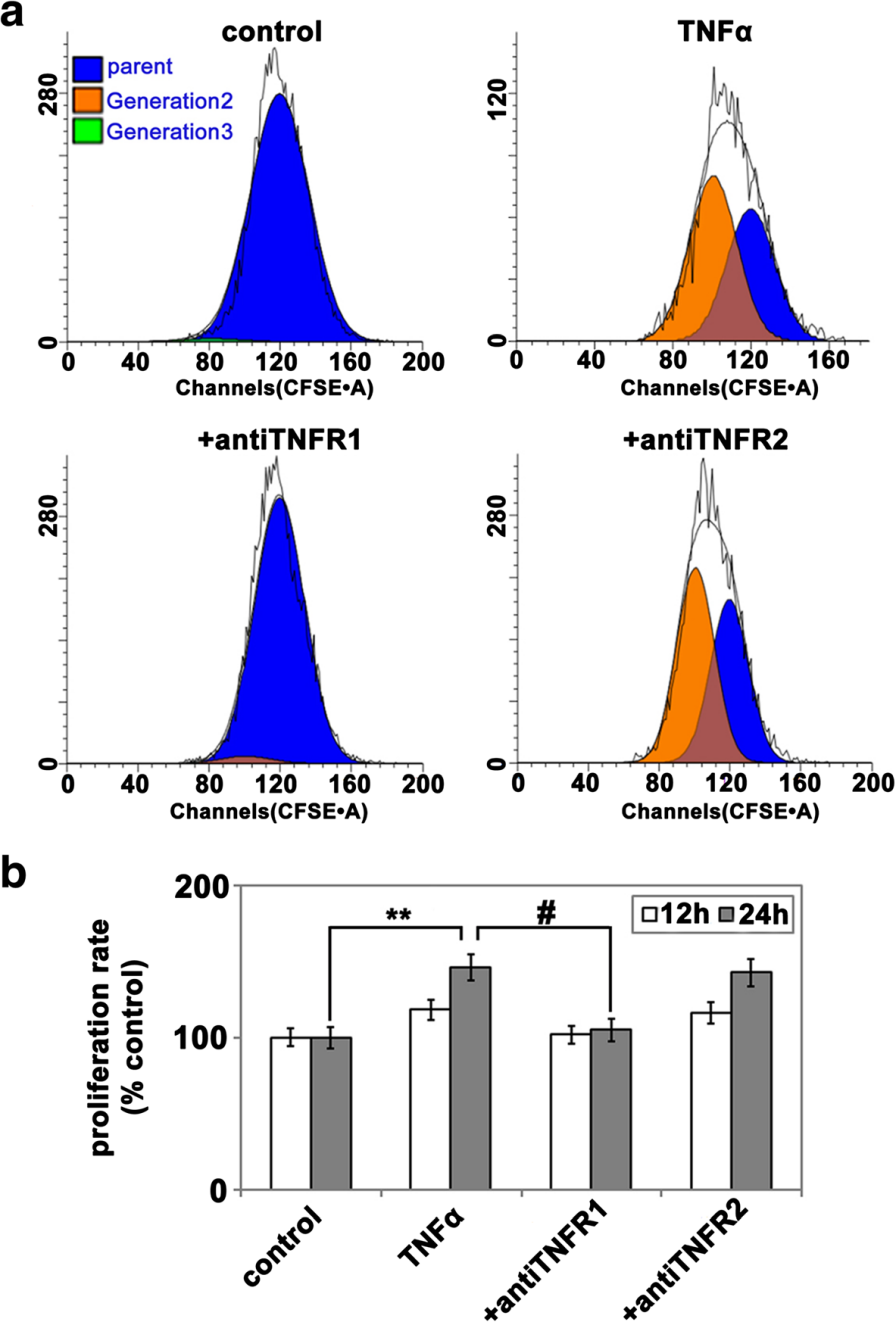
TNF-α promotes HBMEC proliferation through TNFR1, but not TNFR2. CFSE-labeled HBMECs were pre-incubated with 10 μg/ml neutralizing antibodies to TNFR1 or TNFR2 for 30 min and then stimulated with or without 10 ng/ml rhTNF-α. The cultures were incubated for 12 or 24 h before being harvested and analyzed for cell proliferation by flow cytometry. **a** Histogram of HBMEC proliferation with indicated treatments analyzed by ModFit software. **b** Quantification of HBMEC proliferation. The proliferation index was quantified using ModFit software and expressed as the percentage of the control. The results represent the mean ± SEM of four experiments. Note that TNF-α significantly increased HBMEC proliferation relative to control conditions (**P < 0.01); and this was abrogated by the anti-TNFR1 but not the anti-TNFR2 antibody. #*P <* 0.05

As our in vivo studies showed that neutralization of TNFR1 attenuated the post-ischemic angiogenic response, we next examined in vitro whether TNFR1 is directly involved in mediating TNF-α-induced endothelial proliferation. Primary cultures of human brain microvascular endothelial cells (HBMECs) were treated with neutralizing antibodies specific for TNFR1 or TNFR2 prior to stimulation with rhTNF-α before cell proliferation was quantified by FACS CantoII flow cytometry (FCM) and analyzed by ModFit software as described in “Methods”. As shown in Fig. 3, blockade of TNFR1 but not TNFR2 inhibited TNF-α-induced HBMEC proliferation, suggesting that TNFR1 is responsible for mediating TNF-α-induced HBMEC proliferation.

### TNF-α-induced upregulation of α5β1 and αVβ3 integrin expression on HBMECs is mediated through TNFR1 not TNFR2

**Fig. 4:**
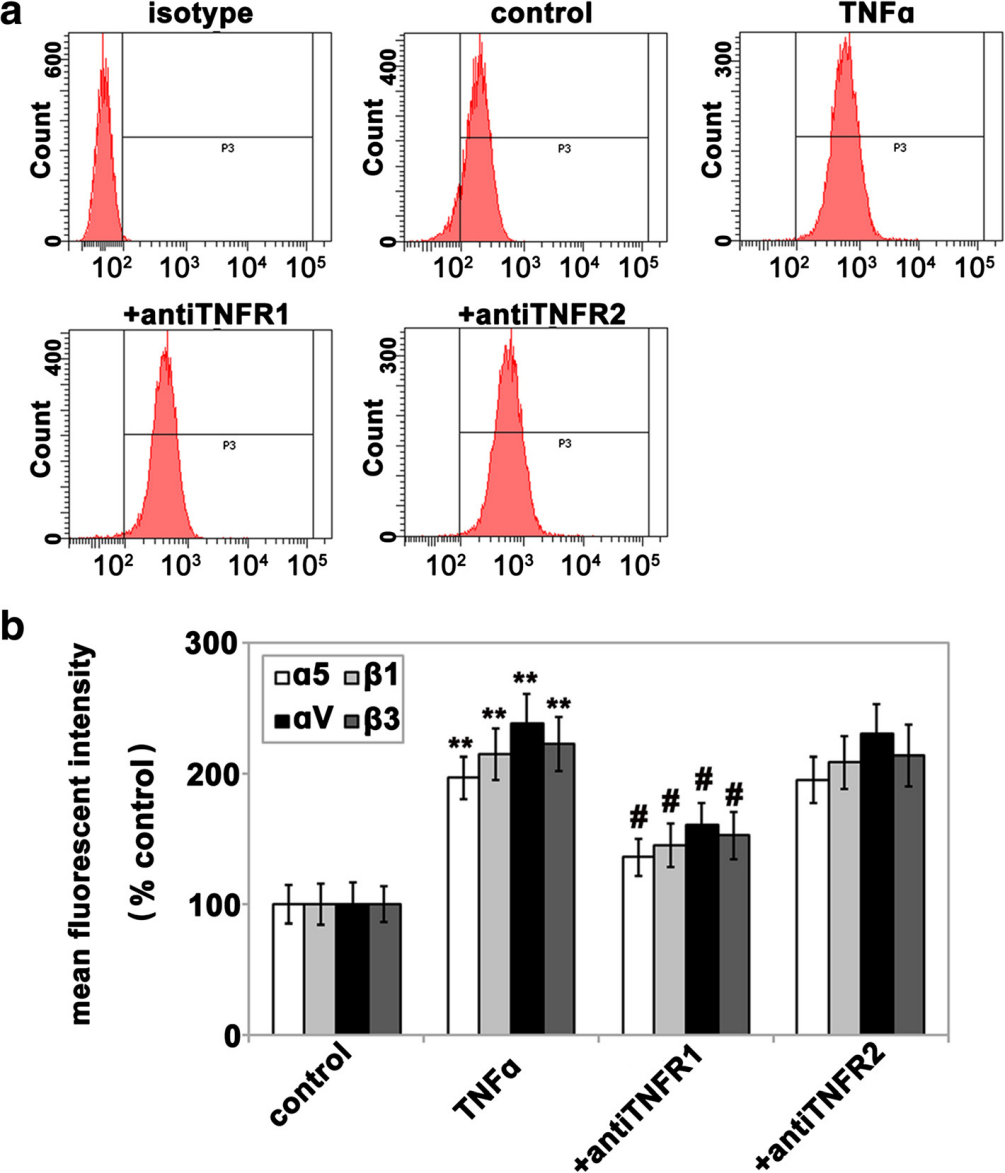
TNF-α-induced upregulation of HBMEC integrins is mediated through TNFR1, but not TNFR2. The HBMECs were pre-incubated with 10 μg/ml neutralizing antibodies to TNFR1 or TNFR2 for 30 min and then stimulated with or without 10 ng/ml rhTNF-α. The cultures were incubated for 12 or 24 h before being harvested and analyzed for expression of α5β1 and αVβ3 integrins by flow cytometry (**a**). Integrin subunit α5 is shown in (**a**). **b** Quantification of α5β1 and αVβ3 integrin expression on HBMECs. For each integrin subunit, the mean fluorescent intensity (MFI) was measured and expression shown as a percentage of the control values. All points represent the mean ± SEM of four experiments. Note that TNF-α significantly increased HBMEC expression of the α5β1 and αVβ3 integrins relative to control (**P < 0.01), and this effect was abrogated by the anti-TNFR1 antibody but not the anti-TNFR2 antibody. #*P <* 0.05

We have previously shown that TNF-α stimulates upregulation of α5β1 and αVβ3 integrins on HBMECs [28]. To determine which TNF receptor mediates this effect, HBMECs were treated with antibodies specifically against TNFR1 or TNFR2 prior to stimulation with rhTNF-α, and 24 h later, α5β1 and αVβ3 integrin expression was analyzed by flow cytometry. As shown in Fig. 4, compared to control conditions, rhTNF-α significantly increased HBMEC expression of the α5, β1, αV, and β3 integrin subunits (*P <* 0.01), and this effect was significantly inhibited by the anti-TNFR1 but not the anti-TNFR2 blocking antibody (*P <* 0.05). This data strongly suggests that TNFR1 mediates TNF-α-induced upregulation of HBMEC α5β1 and αVβ3 integrin expression.

### Signaling through TNFR1 but not TNFR2 mediates the upregulation of endothelial α5β1 and αVβ3 integrins after cerebral ischemia

**Fig. 5:**
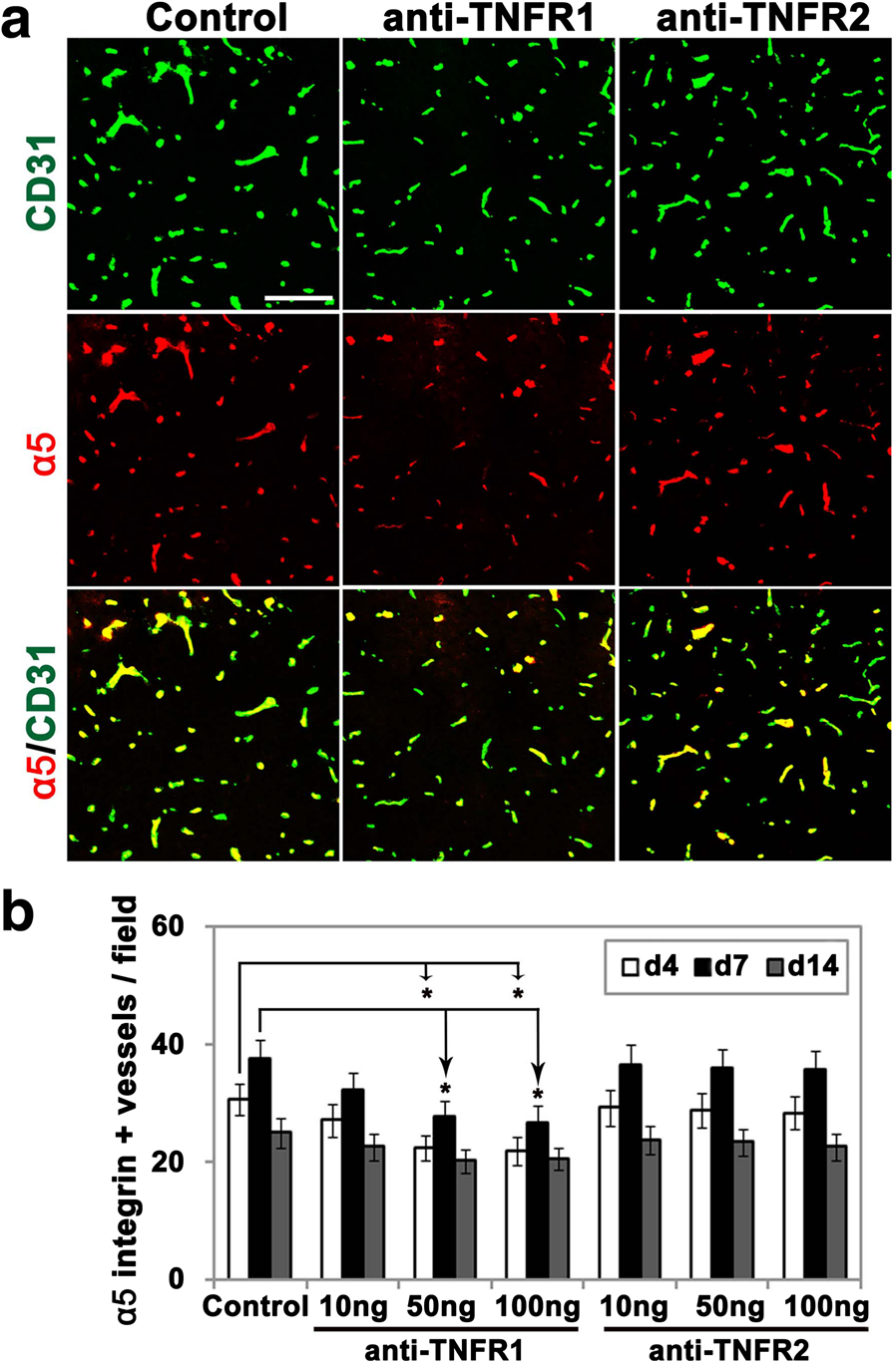
Pharmacological blockade of TNFR1, but not TNFR2, reduced vascular expression of α5 integrin in the cerebral ischemic penumbra. Frozen sections of ischemic penumbra taken from mice after 4, 7, or 14 days reperfusion following 90 min MCAO and having received daily i.c.v. injections of antibodies against TNFR1 or TNFR2 or control IgG, were dual-stained with antibodies against CD31 (AlexaFluor-488) and α5 integrin (Cy-3). **a** After 4 days reperfusion following MCAO, vascular expression of the α5 integrin in the ischemic penumbra of mice receiving the TNFR1 but not TNFR2 antibody (at dose of 50 ng/day) was significantly lower than control mice. *Scale bar* = 100 μm. **b** Quantification of α5 integrin-positive vessels, in which each experiment was performed with five different animals at different time-points post-ischemia. Results are expressed as the mean ± SEM of the number of α5 integrin-positive vessels per FOV. Note that the anti-TNFR1 antibody (at doses of 50 and 100 ng/day), but not the anti-TNFR2 antibody, significantly reduced endothelial expression of the α5 integrin in the ischemic penumbra at 4 and 7 days post-ischemia. *P < 0.05 versus control

**Fig. 6:**
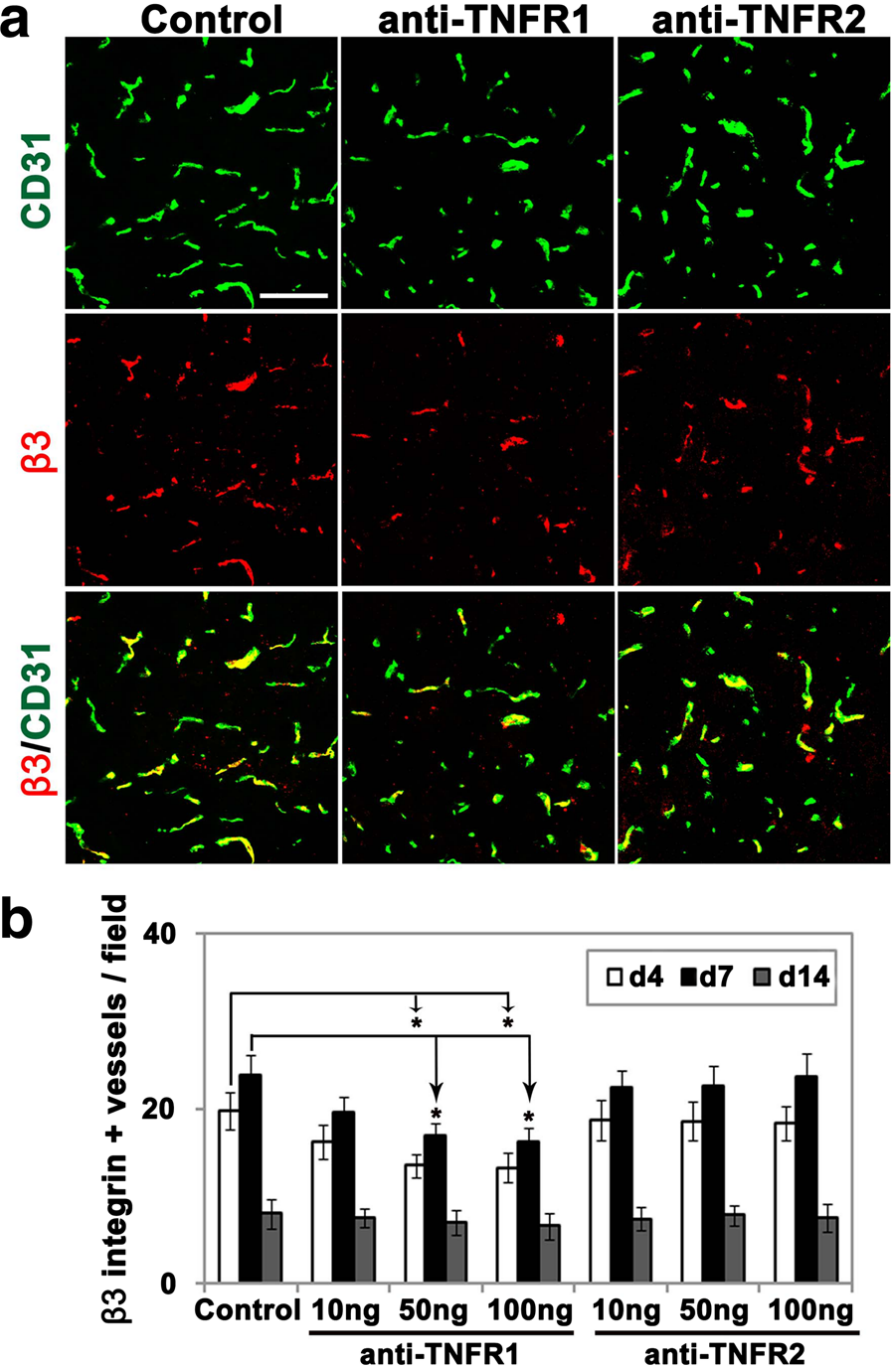
Pharmacological blockade of TNFR1, but not TNFR2, reduced vascular expression of β3 integrin in the cerebral ischemic penumbra. Frozen sections of ischemic penumbra taken from mice after 4, 7, or 14 days reperfusion following 90 min MCAO and having received daily i.c.v. injections of antibodies against TNFR1 or TNFR2 or control IgG, were dual-stained with antibodies against CD31 (AlexaFluor-488) and β3 integrin (Cy-3). **a** After 4 days reperfusion following MCAO, vascular expression of the β3 integrin in the ischemic penumbra of mice receiving the TNFR1 but not TNFR2 antibody (at dose of 50 ng/day) was significantly lower than control mice. *Scale bar* = 100 μm. **b** Quantification of β3 integrin-positive vessels, in which each experiment was performed with five different animals at different time-points post-ischemia. Results are expressed as the mean ± SEM of the number of β3 integrin-positive vessels per FOV. Note that the anti-TNFR1 antibody (at doses of 50 and 100 ng/day), but not the anti-TNFR2 antibody, significantly reduced endothelial expression of β3 integrin in the ischemic penumbra at 4 and 7 days post-ischemia. *P < 0.05 versus control

In prior studies, we have demonstrated that cerebral ischemia induces upregulation of vascular fibronectin, and its two endothelial receptors α5β1 and αVβ3 integrins [9]. As our in vitro studies above suggest that TNF-α induction of α5β1 and αVβ3 integrins is mediated through the TNFR1 pathway, we next examined this process in vivo. According to the two-way repeated-measures ANOVA, the effect of treatment (antibodies against TNFR1, TNFR2, or control IgG) and time (day 4, 7, or 14) were highly significant for both α5 (treatment: DF = 6; *F =* 4.17; *P <* 0.01; time: DF = 2; *F =* 27.28; *P <* 0.001) and β3 (treatment: DF = 6; *F =* 3.84; *P <* 0.01; time: DF = 2; *F =* 98.19; *P <* 0.001) integrin-positive vessels. The interaction of the variables “time and treatment” was not significant for both α5 (DF = 12; *F =* 0.29; *P =* 0.99) and β3 (DF = 12; *F =* 0.66; *P =* 0.78) integrin-positive vessels. As shown in Figs. 5 and 6, the Bonferroni post hoc test showed that after 4 or 7 days reperfusion following MCAO, the number of α5 and β3 integrin-positive vessels in the ischemic penumbra of mice receiving the TNFR1 antibody (at doses of 50 and 100 ng/day) was significantly lower than control mice (for the 50 ng group: 22.3 ± 2.2 vs. 30.6 ± 2.7, *P <* 0.05 at day 4 for α5, 13.4 ± 1.4 vs. 19.7 ± 2.1, *P <* 0.05 at day 4 for β3, 27.6 ± 2.8 vs. 37.7 ± 3.2, *P <* 0.05 at day 7 for α5, 16.9 ± 1.4 vs. 23.8 ± 2.4, *P <* 0.05 at day 7 for β3; for the 100 ng group: 21.9 ± 2.4 vs. 30.6 ± 2.7, *P <* 0.05 at day 4 for α5, 13.2 ± 1.6 vs. 19.7 ± 2.1, *P <* 0.05 at day 4 for β3, 26.5 ± 3 vs. 37.7 ± 3.2, *P <* 0.05 at day 7 for α5, 16.1 ± 1.6 vs. 23.8 ± 2.4, *P <* 0.05 at day 7 for β3). The number of α5/β3 integrin-positive vessels under all conditions had reduced to almost baseline levels 14 days following reperfusion. In contrast to the TNFR1 antibody, the TNFR2 antibody had no appreciable effect on the number of α5/β3 integrin-positive vessels. These results suggest that signaling through TNFR1 but not TNFR2 is responsible for the upregulation of α5β1 and αVβ3 integrins on brain endothelial cells following cerebral ischemia.

### Neutralization of α5β1 and αVβ3 integrins blocks TNF-α-induced HBMEC proliferation

**Fig. 7:**
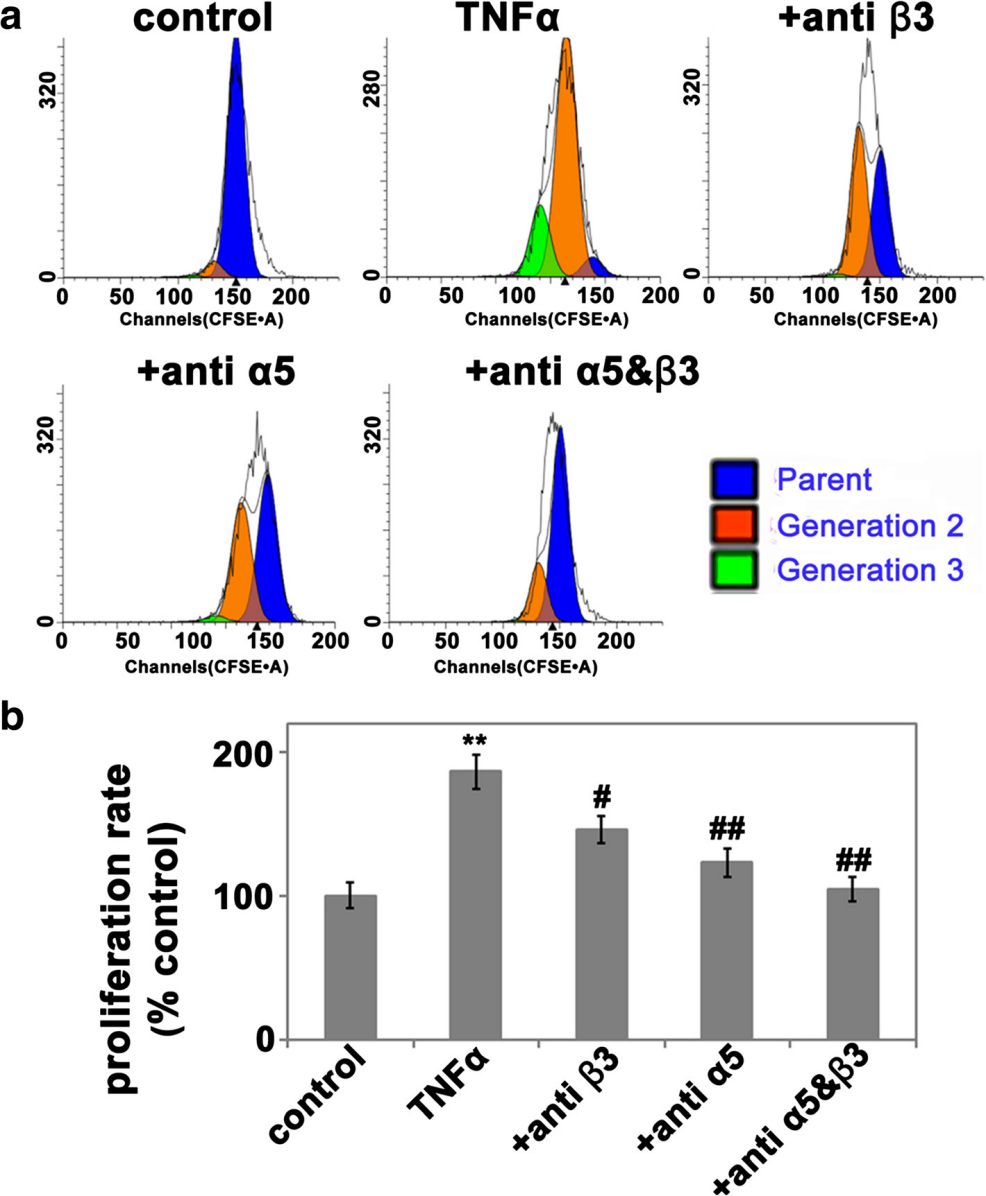
TNF-α-induced HBMEC proliferation was abrogated by antibody neutralization of the α5 or αVβ3 integrins. CFSE-labeled HBMECs were pre-incubated with neutralizing antibodies (5 μg/ml) against the human integrin α5 or αVβ3 integrins, or a combination of both for 30 min and then stimulated with or without 10 ng/ml rhTNF-α for an additional 24 h before being harvested and analyzed for cell proliferation by flow cytometry. **a** Histogram of HBMEC proliferation with indicated treatments analyzed by ModFit software. **b** Quantification of HBMEC proliferation. The proliferation index was quantified using ModFit software and expressed as the percentage of the control. The results represent the mean ± SEM of four experiments. Note that TNF-α significantly increased HBMEC proliferation relative to control conditions (**P < 0.01); and this was abrogated by either anti-α5, anti-αVβ3, or a combination of both antibodies. #*P <* 0.05, ##*P <* 0.01 versus positive control

Our findings have demonstrated that TNF-α induces parallel increases in HBMEC proliferation and expression of α5β1 and αVβ3 integrins. In our previous in vivo study, we found that vascular expression of fibronectin and theα5 and β3 integrins in the ischemic penumbra were all significantly increased by day 4, peaked at day 7, then declined at day 14. Interestingly, brain endothelial cell proliferation follows exactly the same time-course, suggesting a mechanistic relationship, with the Fn-α5β1/αVβ3 integrin axis driving brain endothelial cell proliferation and angiogenesis [9]. These results indicate that the brain endothelial cell proliferation induced by TNF-α is at least partially due to the increased surface expression of the integrins. If this is true, one would expect to see attenuated brain endothelial cell proliferation when the α5β1 and αVβ3 functions are blocked. To test this hypothesis, HBMEC proliferation was performed in the presence or absence of function-blocking antibodies to α5β1, αVβ3, or a combination of both integrins prior to stimulation with rhTNF-α. As shown in Fig. 7, HBMEC proliferation was significantly inhibited by the α5- or αVβ3-blocking antibodies (*P <* 0.01, or *P <* 0.05 vs. control) and further reduced by the combination of α5 and αVβ3 antibodies together (*P <* 0.01). Based on these results, we conclude that both α5β1 and αVβ3 integrins play an important role in TNF-α-induced endothelial cell proliferation.

## Discussion

A role for TNF-α in ischemic tissue injury and neurotoxicity is well known [33], whereas the role of the two distinct TNF-α receptors in mediating angiogenic signaling after cerebral ischemic stroke is relatively unexplored. In this study, we defined which TNF receptor is involved in promoting angiogenesis after cerebral ischemia and also examined the role of the downstream angiogenic integrins α5β1 and αVβ3 in mediating this process. Our main findings were as follows: (i) blockade of TNFR1 but not TNFR2 reduced post-ischemic angiogenic remodeling, as shown by reduced endothelial proliferation and reduced vascular density in the ischemic penumbra, (ii) blockade of TNFR1 but not TNFR2 also reduced expression of the α5β1 and αVβ3 integrins on blood vessels in the ischemic penumbra, (iii) TNF-α stimulation of HBMEC cell proliferation and α5β1 and αVβ3 integrin expression is mediated through TNFR1 but not TNFR2, and (iv) α5β1 and αVβ3 integrin signaling is required for TNF-α-induced HBMEC proliferation. Taken together, these data highlight an important role for TNFR1 in orchestrating vascular remodeling events in the ischemic penumbra following cerebral ischemia, and point to the endothelial integrins α5β1 and αVβ3 as key mediators of this response.

### Signaling through TNFR1 mediates angiogenesis after cerebral ischemia

Several studies suggest that the neuroprotective effect of TNF-α following stroke is mediated through TNFR1 [18, 34–37]. Taoufik and colleagues found that after focal cerebral ischemia, TNF protects by activating the NF-kB-FLIPL signaling pathway downstream of TNFR1, and it can also act directly on endothelial cells to induce angiogenesis through activation of the well-known angiogenic factor vascular endothelial growth factor (VEGF) [38, 39]. Consistent with these reports, the work presented here shows that blocking signaling via TNFR1 but not TNFR2 early following cerebral ischemia suppressed the ensuing angiogenic response. In parallel in vitro studies, TNF-α induction of brain endothelial cell proliferation was abrogated by antibody blockade of TNFR1 but not TNFR2, further supporting the concept that TNF-α promotes angiogenesis through TNFR1, not TNFR2.

Interestingly, outside the CNS, in a model of hindlimb ischemia, it has been reported that TNFR1 inhibits, but TNFR2 signaling promotes, TNF-α-induced arteriogenesis and angiogenesis [24]. Furthermore, in a myocardial infarct model, TNFR1-mediated signaling has a deleterious effect while TNFR2-mediated signaling is protective and promotes repair and regeneration processes [40]. The molecular basis for these apparent differences is currently unclear, but possible explanations might be related to organ-specific microenvironments or pathological models used, and it is well known that the angiogenic effect of TNF-α varies with different cell lines and experimental conditions [41–43]. Another reason may relate to the degree of ischemia employed. Our model of MCAO is only temporary (90 min), but the hindlimb ischemia model represents a permanent state of severe ischemia, and it is possible that TNFR2 signaling is activated only in response to severe ischemia [24]. Alternatively, it is worth considering that the hindlimb ischemia and MI models used genetic mouse KOs of TNFR1 and TNFR2, while our studies employed the use of neutralizing antibodies [24, 40].

### Signaling through TNFR1 is required for upregulation of α5β1 and αVβ3 integrins on brain endothelial cells after cerebral ischemia

TNF-α has been shown to significantly increase both the cell surface and total cellular expression of αVβ3 integrins on bovine pulmonary artery endothelial cells [44] as well as induce β1 activation and significantly increase α5β1 integrin expression on the surface of human umbilical vein endothelial cells (HUVECS) [45]. This is consistent with our earlier work showing that TNF-α stimulates upregulation of α5β1 and αVβ3 integrin expression on HBMECs [28]. However, it was previously unknown which TNFR mediated this effect. Our present study provides strong evidence that signaling through TNFR1 but not TNFR2 is essential for the upregulation of both α5β1 and αVβ3 integrins on brain endothelial cells after cerebral ischemia.

### α5β1 and αVβ3 integrins are critical for TNF-α-induced cell proliferation

Extracellular matrix (ECM) proteins play an essential role in angiogenesis. In particular, Fn and its integrin receptors are indispensable, as shown by failure of angiogenesis in mutant mice lacking Fn [46] or the Fn-specific receptor, α5β1 integrin [47]. In previous studies, we showed that cerebral hypoxia and cerebral ischemia induce a strong angiogenic response in the brain, which correlates with induction of fibronectin and its two receptors, α5β1 and αVβ3 integrins on angiogenic cerebral vessels [5, 6, 9, 48, 49]. Interestingly, in both these models, BEC proliferation followed exactly the same time-course as the upregulated expression of fibronectin and its receptors, the α5β1 and αVβ3 integrins on blood vessels [9]. Furthermore, in the chronic hypoxia model, we found that transgenic mice lacking endothelial expression of α5 integrin (α5-EC-KO mice) showed a delayed and reduced angiogenic response to mild hypoxia [6]. Taken together, these results indicate that the fibronectin-α5β1/αVβ3 integrin axis drives BEC proliferation and angiogenesis following cerebral hypoxia or ischemia.

## Conclusions

The aim of this study was to define which TNF receptor is involved in promoting angiogenesis after cerebral ischemia and investigate the potential role of the endothelial integrins α5β1 and αVβ3 in mediating this process. Our results suggest that TNFR1, but not TNFR2-mediated signaling, plays a critical role in promoting angiogenic responses following cerebral ischemia and, furthermore, that the angiogenic integrins α5β1 and αVβ3 are important mediators of this effect. These novel findings could form the basis for future therapeutic strategies aimed at stimulating angiogenesis following cerebral ischemia.

## Additional file

Additional file 1: TTC staining differentiates the infarct (white) from viable tissue (red). The brain sections taken from mice after 4 days reperfusion following 90 min MCAO and having received daily i.c.v. injections of antibodies against TNFR1 or TNFR2 or control IgG were stained with 2 % TTC. Note that the mice receiving TNFR1 antibody (at doses of 50 and 100ng/day) showed larger infarct size than mice receiving TNFR2 antibody or control antibody. (JPG 455 kb)

## Abbreviations

BEC: Brain endothelial cell
bFGF: Basic fibroblast growth factor
CFSE: Carboxy-fluorescein succinimidyl ester
EBM: Endothelial basal medium
ECM: Extracellular matrix
FCM: Flow cytometry
Fn: Fibronectin
FOV: Positive events per field of view
HBMECs: Human brain microvascular endothelial cells
IF: Immunofluorescent
MCAO: Occlusion of the middle cerebral artery
NIH: National Institutes of Health
rhTNF: Recombinant human tumor necrosis factor
TNF-α: Tumor necrosis factor-α
TNFR1: Tumor necrosis factor receptor 1
VEGF: Vascular endothelial growth factor
