# Supplementary figures and images for: Cerebral ischemia-induced angiogenesis is dependent on tumor necrosis factor receptor 1-mediated upregulation of α5β1 and αVβ3 integrins
Source: J Neuroinflammation. 2016 Sep 1;13(1):227. doi: 10.1186/s12974-016-0697-1 (PMC5009537; doi:10.1186/s12974-016-0697-1)

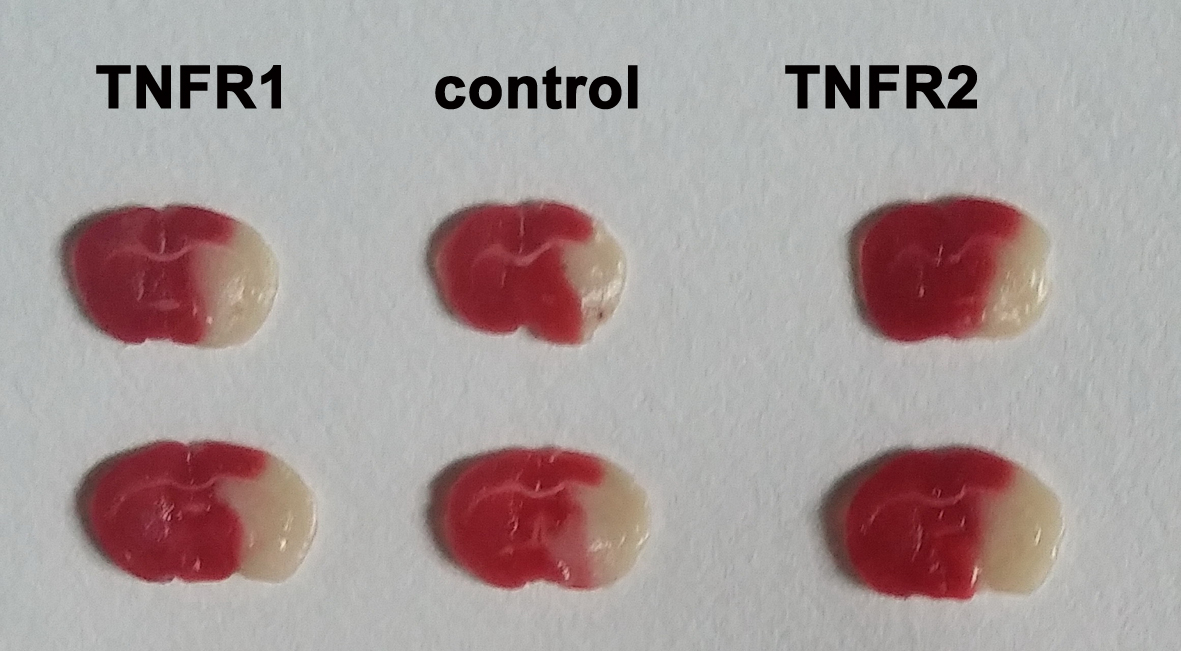

Supplement: Additional file 1: — TTC staining differentiates the infarct (white) from viable tissue (red). The brain sections taken from mice after 4 days reperfusion following 90 min MCAO and having received daily i.c.v. injections of antibodies against TNFR1 or TNFR2 or control IgG were stained with 2 % TTC. Note that the mice receiving TNFR1 antibody (at doses of 50 and 100ng/day) showed larger infarct size than mice receiving TNFR2 antibody or control antibody. (JPG 455 kb) [file 12974_2016_697_MOESM1_ESM.jpg]
